# Establishment and validation of a prognostic nomogram for postoperative patients with gastric cardia adenocarcinoma: A study based on the Surveillance, Epidemiology, and End Results database and a Chinese cohort

**DOI:** 10.1002/cam4.5994

**Published:** 2023-05-03

**Authors:** Lei Wang, Jingjing Ge, Liwen Feng, Zehua Wang, Wenjia Wang, Huiqiong Han, Yanru Qin

**Affiliations:** ^1^ Department of Oncology The First Affiliated Hospital of Zhengzhou University Zhengzhou People's Republic of China

**Keywords:** gastric cardia adenocarcinoma, LODDS, nomogram, prognosis, SEER database

## Abstract

**Background:**

Gastric cardia adenocarcinoma (GCA) is a highly fatal form of cancer in humans. The aim of this study was to extract clinicopathological data of postoperative patients with GCA from the Surveillance, Epidemiology, and End Results database, analyze prognostic risk factors, and build a nomogram.

**Methods:**

In this study, the clinical information of 1448 patients with GCA who underwent radical surgery and were diagnosed between 2010 and 2015 was extracted from the SEER database. The patients were then randomly divided into training (*n* = 1013) and internal validation (*n* = 435) cohorts at a 7:3 ratio. The study also included an external validation cohort (*n* = 218) from a Chinese hospital. The study used the Cox and LASSO models to pinpoint the independent risk factors linked to GCA. The prognostic model was constructed according to the results of the multivariate regression analysis. To assess the predictive accuracy of the nomogram, four methods were used: C‐index, calibration curve, time‐dependent ROC curve, and DCA curve. Kaplan–Meier survival curves were also generated to illustrate the differences in cancer‐specific survival (CSS) between the groups.

**Results:**

The results of the multivariate Cox regression analysis showed that age, grade, race, marital status, T stage, and log odds of positive lymph nodes (LODDS) were independently associated with cancer‐specific survival in the training cohort. Both the C‐index and AUC values depicted in the nomogram were greater than 0.71. The calibration curve revealed that the nomogram's CSS prediction was consistent with the actual outcomes. The decision curve analysis suggested moderately positive net benefits. Based on the nomogram risk score, significant differences in survival between the high‐ and low‐risk groups were observed.

**Conclusions:**

Race, age, marital status, differentiation grade, T stage, and LODDS are independent predictors of CSS in patients with GCA after radical surgery. Our predictive nomogram constructed based on these variables demonstrated good predictive ability.

## Abstract 2

The nomogram for postoperative patients with GCA we constructed has good predictive power and can help clinicians accurately assess patient prognosis and identify high‐risk patients to develop more personalized treatment plans.

## 1 INTRODUCTION

Gastric cardia adenocarcinoma (GCA) is a commonly diagnosed malignant tumor of the digestive tract.^1^ Although the overall incidence of gastric cancer (GC) has recently declined globally, the incidence of GCA is still increasing.^1^, ^2^ GCA originates at the independent zone of the esophagogastric junction (EGJ), which differs from GC that originates at other sites in terms of pathophysiology.^3^ GCA remains a challenging disease, and radical surgery is currently the only available management method. Early diagnosis and timely surgery can have a positive impact on the prognosis of patients with GCA. In addition, despite advances in treatment, the prognosis for patients with GCA remains suboptimal due to factors such as tumor recurrence.^4^, ^5^ However, the introduction of personalized treatment has directed renewed attention to the prognostic factors that impact patients with cancer. Therefore, it is significant to analyze the prognostic risk factors and construct a survival prediction model for patients with GCA after radical surgery.

The TNM staging system is currently the primary method used to evaluate patient prognosis and guide clinical treatment approaches.^6^ However, relying solely on the TNM staging system has obvious limitations, as it lacks important variables such as age, differentiation grade, and other influential factors. Therefore, it is not suitable for individualized analysis.^7^, ^8^ Multiple studies have demonstrated that the log odds of positive lymph nodes (LODDS) are superior to the N stage alone in determining lymph node metastasis and providing accurate prognostic assessments for patients with cancer.^9^, ^10^ In line with this, a nomogram is a graphical calculation instrument based on statistical models that can predict the likely outcomes.^11^ Each variable is assigned a score based on its degree of risk, and the final sum of all scores corresponds to the predicted survival probability. Prior research has demonstrated that nomograms have a better prediction performance compared to that of TNM staging, leading to the construction of various nomograms to predict prognosis in different types of cancer.^12^, ^13^, ^14^, ^15^

The Surveillance, Epidemiology, and End Results (SEER) database covers over 28% of the population of the United States and includes information on patient demographics, stage of diagnosis, treatment process, tumor morphology, primary tumor site, vital status follow‐up, and causes of death, thus providing an effective tool for tumor epidemiological research.^16^ The objective of this study was to obtain clinical data from the SEER database and use it to analyze the prognostic risk factors in postoperative patients with GCA. The study also aimed to construct a nomogram model that could accurately predict cancer‐specific survival (CSS) in patients with GCA and evaluate the nomogram through internal and external validation.

## 2 METHODS

### 2.1 Patient selection

The specific operational process for extracting information from the SEER database is outlined below: (1) Register for a personal account on the SEER database official website and install the SEER*Stat software; (2) Extract clinical data of patients based on the inclusion and exclusion criteria determined in this study; (3) Export the extracted data as a spreadsheet and proceed to the next step of organizing and analyzing the data.

**FIGURE 1:**
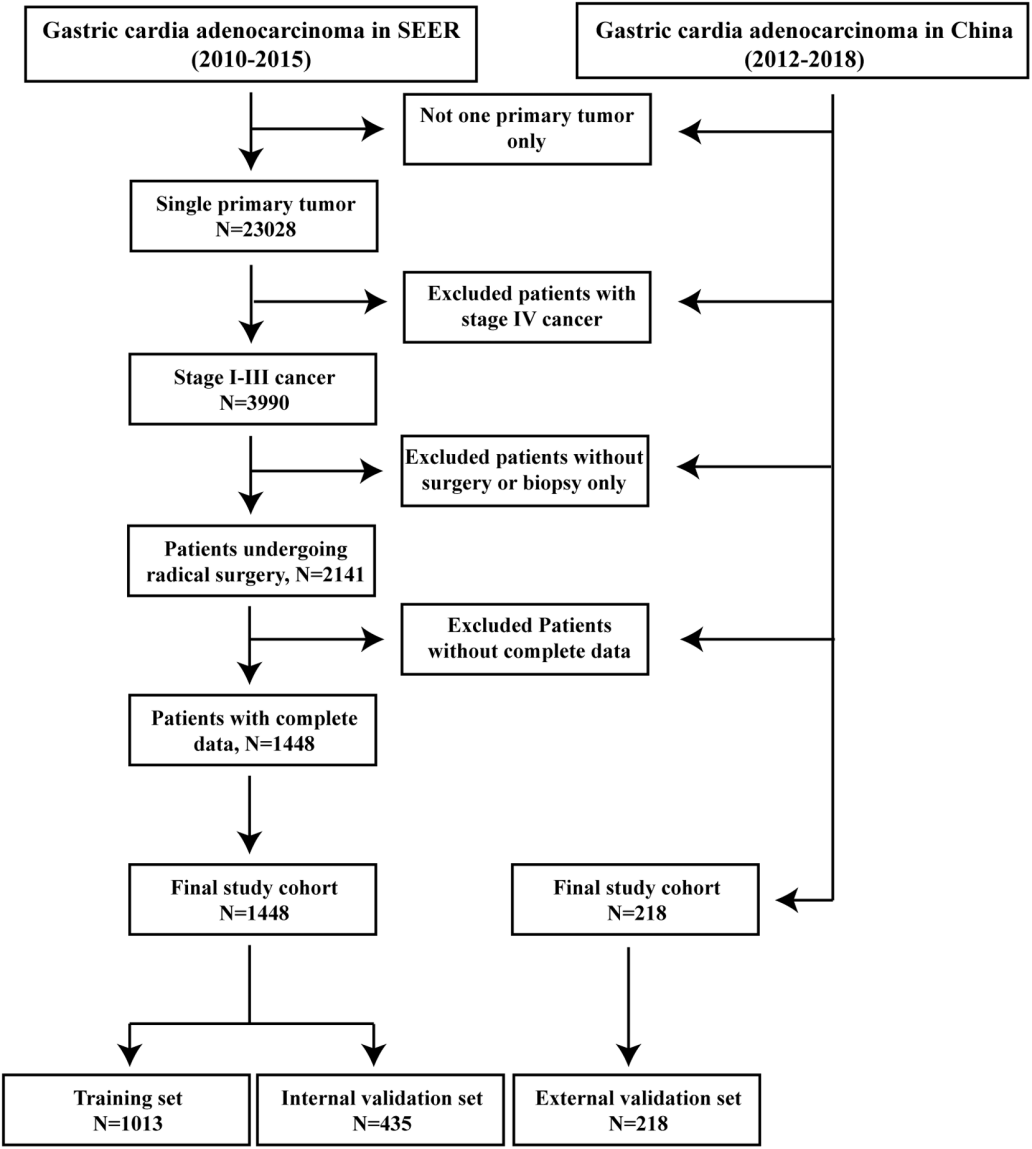
Flowchart for the selection of the patients.

The data for patients with GCA between 2010 and 2015 were downloaded from the SEER*Stat 8.4.0 software using a private ID (13914‐Nov2021), based on the 2021 release of the SEER database. According to the SEER codes, patients with adenocarcinoma (ICD‐O‐3 codes: 8140–8145,8147, 8210, 8211, 8214, 8220, 8221, 8230, 8231, 8255, 8260–8263, 8310, 8480, 8481, 8490, 8510, 8560, 8562, 8570–8576) and tumors located in the cardia (site code: C16.0) were included. All patients underwent radical surgery (surgery encode 30–80); however, those with stage IV GCA were excluded due to the controversial nature of their operation. In addition, patients with missing information on relevant variables were excluded from this study. The detailed screening process and selection criteria are shown in Figure 1. The patients included in this study were randomly divided into training (70%) and internal validation (30%) groups.

In addition to the above data, the data of patients with GCA after surgery at the First Affiliated Hospital of Zhengzhou University between 2012 and 2018 were collected for validation. Patients underwent thorough preoperative assessments, including physical exams, medical history recording, hematological testing, and imaging. Furthermore, tumor metastasis was evaluated using various imaging techniques. The study was approved by the hospital ethics committee, and patient records were kept confidential in accordance with ethical standards.

### 2.2 Variable collection

According to the instructions provided by the National Cancer Institute, the code data extracted from the SEER database was translated. The external validation cohort data was obtained through the hospital medical record system. The variables collected in this study were mainly divided into three categories: patient‐related variables, disease‐related variables, and follow‐up information. (1) Patient‐related variables: race, sex, age at diagnosis, and marital status. (2) Disease‐related variables: histological type, differentiation grade, 7th edition AJCC clinical stage (TNM), tumor size, examined lymph nodes (ELN), positive lymph nodes (PLN), and LODDS, which is calculated as LODDS = log[(PLN + 0.5)/(ELN‐PLN + 0.5)]. (3) Follow‐up information: survival status, cause of death, and survival time.

Each variable was categorized as follows: marital status, unmarried or married; sex, male or female; histological type, adenocarcinoma or signet ring cell carcinoma; differentiation grade, G1–2 (well to moderately differentiated) or G3–4 (poorly differentiated and undifferentiated); and the 7th edition AJCC clinical stage, which divided T stage into T1, T2, T3, and T4 and N stage into N0, N1, N2, and N3. The X‐tile software was used to determine the optimal cutoff values for the three continuous variables of age, tumor size, and LODDS. Age was classified as <65, 65–71, and >71 years; tumor size was classified as <2.6, 2.6–4.8, and <4.8 cm; LODDS was classified as LODDS1 (< −1.20), LODDS2 (−1.20 to −0.60), and LODDS3 (< −0.60).

### 2.3 Statistical analysis

SPSS (26.0) and R (4.2.2) software were used for statistical analysis and graph plotting. The X‐tile software determined the optimal cutoff value, and continuous variables were transformed into categorical variables accordingly. Categorical variables were compared between groups using the Fisher's exact test or chi‐square test. Univariate Cox analysis was conducted for each variable, and variables with statistical significance (*p* < 0.05) were included in the LASSO equation for feature selection. In addition, the multivariate Cox analysis was performed to identify independent predictive factors. A two‐tailed *p* < 0.05 was considered statistically significant.

Based on the multivariate Cox regression analysis, a nomogram was constructed to predict patient CSS using the rms and survival packages in R software. The model's reliability was validated by internal and external validation cohorts. The model's discrimination was evaluated using the concordance index (C‐index), with a C‐index greater than 0.71 indicating excellent discrimination. The calibration curve measured the degree of closeness between the predicted and actual risk, with a closer curve indicating better predictive results. The time‐dependent receiver operating characteristic curve (ROC curve) evaluated the model's accuracy, with an area under the curve (AUC) greater than 0.71 indicating good predictive ability. Decision curve analysis (DCA) evaluated the model's clinical utility and quantified the net benefits at different threshold probabilities. Patients were divided into high‐risk and low‐risk groups based on the median risk score from the column chart. Kaplan–Meier analysis was used to plot the survival curves of high‐risk and low‐risk groups for predicting CSS, and the log‐rank test was used for survival analysis.

## 3 RESULTS

### 3.1 Patient characteristics

Data of 1448 patients with GCA who underwent curative surgery were extracted from the SEER database, and these patients were randomly assigned to a training set (1013 patients) and an internal validation set (435 patients) at a ratio of 7:3. In the training cohort, the median follow‐up time was 41 months, with 3‐year and 5‐year CSS rates of 57.7% and 47.3%, respectively. In the internal validation cohort, the median follow‐up time was 37 months, with 3‐year and 5‐year CSS rates of 55.5% and 43.9%, respectively. In the training cohort, there were 818 male (80.8%) and 195 female (19.2%) patients. Of the total sample, 86.2% (*n* = 873) were White, 4.6% (*n* = 47) were Black, and 9.2% (*n* = 93) belonged to other racial categories. Overall, 555 patients aged <65 years (54.8%), 241 aged between 65 and 71 years (23.8%), and 217 aged >71 years (21.4%). In the internal validation cohort, there were 366 male (84.1%) and 69 female (15.9%) patients. Of the total sample, 88.1% (*n* = 383) were White, 4.8% (*n* = 21) were Black, and 7.1% (*n* = 31) belonged to other racial categories. In this cohort, 230 patients aged <65 years (52.9%), 97 aged between 65 and 71 years (22.3%), and 108 aged >71 years (24.8%). The overall grouping of the training and internal validation cohorts was consistent with simple random grouping.

Retrospective clinical data of 218 patients with GCA who underwent curative surgery at a Chinese hospital were collected, and these patients were assigned to an external validation cohort according to the same inclusion and exclusion criteria, and the nomogram model was validated with external data. In the external validation cohort, the median follow‐up time was 31 months, with 3‐year and 5‐year CSS rates of 69.0% and 58.6%, respectively. There were 174 male (79.8%) and 44 female (20.2%) patients in the external validation cohort. In this cohort, 136 patients aged <65 years (62.4%), 57 aged between 65 and 71 years (26.1%), and 25 aged >71 years (11.5%). The basic information is presented in Table 1.

**TABLE 1:** Clinicopathological characteristics of the postoperative patients with stage I–III GCA.

Variable	NO. (%)		
	Training cohort	Internal validation cohort	External validation cohort
Sex			
Female	195 (19.2)	69 (15.9)	44 (20.2)
Male	818 (80.8)	366 (84.1)	174 (79.8)
Race			
White	873 (86.2)	383 (88.1)	0 (0)
Black	47 (4.6)	21 (4.8)	0 (0)
Other	93 (9.2)	31 (7.1)	218 (100)
Age, years			
<65	555 (54.8)	230 (52.9)	136 (62.4)
65–71	241 (23.8)	97 (22.3)	57 (26.1)
>71	217 (21.4)	108 (24.8)	25 (11.5)
Marital status			
Unmarried	294 (29.0)	129 (29.7)	7 (3.2)
Married	719 (71.0)	306 (70.3)	211 (96.8)
Histological type			
Adenocarcinoma	907 (89.5)	400 (92.0)	206 (94.5)
Signet ring carcinoma	106 (10.5)	35 (8.0)	12 (5.5)
Tumor grade			
Well	442 (43.6)	189 (43.4)	59 (27.1)
Poor	571 (56.4)	246 (56.6)	159 (72.9)
TNM stage			
I	189 (18.7)	78 (17.9)	14 (6.4)
II	275 (27.1)	114 (26.2)	68 (31.2)
III	549 (54.2)	243 (55.9)	136 (62.4)
T stage			
T1	200 (19.8)	80 (18.4)	8 (3.7)
T2	143 (14.1)	69 (15.9)	20 (9.2)
T3	609 (60.1)	260 (59.8)	37 (16.9)
T4	61 (6.0)	26 (5.9)	153 (70.2)
N stage			
N0	382 (37.7)	157 (36.1)	67 (30.7)
N1	346 (34.2)	136 (31.3)	47 (21.6)
N2	170 (16.8)	86 (19.8)	51 (23.4)
N3	115 (11.3)	56 (12.8)	53 (24.3)
Size, cm			
<2.6	310 (30.6)	144 (33.1)	29 (13.3)
2.6–4.8	364 (35.9)	156 (35.9)	102 (46.8)
>4.8	339 (33.5)	135 (31.0)	87 (39.9)
LODDS			
LODDS1	456 (45.0)	194 (44.6)	67 (30.7)
LODDS2	288 (28.4)	125 (28.7)	46 (21.1)
LODDS3	269 (26.6)	116 (26.7)	105 (48.2)

### 3.2 Independent prognostic factors

**FIGURE 2:**
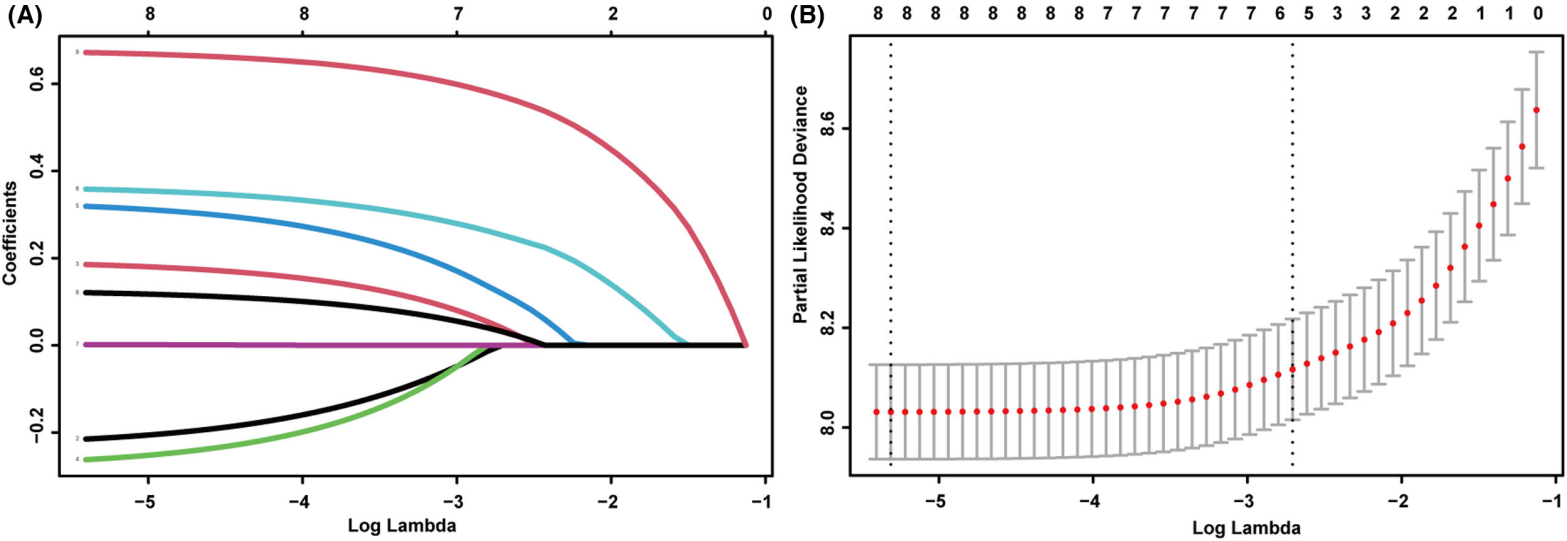
LASSO coefficients of eight features (A) and the selection of tuning parameter (λ) for the LASSO model (B).

The univariate Cox regression analysis showed that race, age, marital status, grade, T stage, N stage, tumor size, and LODDS score were related to CSS. To avoid overfitting, LASSO regression was performed using those eight variables. All eight variables were included in the model, as their coefficients were non‐zero (Figure 2). The variables that showed significance in the univariate and LASSO regression analyses were included in the multivariate analysis. In the multifactorial Cox analysis, age (65–71 and >71 years), grade (poor), T stage (T1–3), and LODDS (LODDS2 and LODDS2) were independent prognostic risk factors in the patients with GCA at stages I–III. Moreover, race (other) and marital status (married) were protective factors that showed better patient prognosis. Table 2 displays the outcomes of both univariate and multivariate Cox analyses conducted on the training set.

**TABLE 2:** Univariate and multivariate cox regression analyses of the prognostic factors for CSS.

Variables	Univariate analysis		Multivariate analysis	
	HR (95% CI)	*p*‐value	HR (95% CI)	*p*‐value
Sex				
Female	1			
Male	0.989 (0.796–1.229)	0.922		
Race				
White	1		1	
Black	1.121 (0.765–1.641)	0.558	0.910 (0.616–1.346)	0.638
Other	0.621 (0.440–0.875)	**0.007**	0.621 (0.439–0.877)	**0.007**
Age, years				
<65	1		1	
65–71	1.240 (1.006–1.527)	**0.043**	1.348 (1.091–1.666)	**0.006**
>71	1.445 (1.168–1.788)	**<0.001**	1.472 (1.184–1.830)	**<0.001**
Marital status				
Unmarried	1		1	
Married	0.787 (0.654–0.946)	**0.011**	0.742 (0.614–0.897)	**0.002**
Histological type				
Adenocarcinoma	1		1	
Signet ring carcinoma	1.096 (0.832–1.443)	0.514		
Tumor grade				
Well	1		1	
Poor	1.809 (1.513–2.163)	**<0.001**	1.405 (1.168–1.689)	**<0.001**
T stage				
T1	1		1	
T2	2.044 (1.402–2.980)	**<0.001**	1.613 (1.091–2.383)	**0.016**
T3	3.569 (2.553–4.800)	**<0.001**	2.304 (1.649–3.218)	**<0.001**
T4	6.029 (4.025–9.032)	**<0.001**	2.868 (1.826–4.505)	**<0.001**
N stage				
N0	1		1	
N1	2.183 (1.735–2.746)	**<0.001**	1.004 (0.758–1.329)	0.979
N2	3.742 (2.907–4.816)	**<0.001**	1.152 (0.820–1.620)	0.415
N3	4.885 (3.703–6.443)	**<0.001**	0.983 (0.661–1.461)	0.931
Size, cm				
<2.6	1		1	
2.6–4.8	1.736 (1.374–2.194)	**<0.001**	1.101 (0.855–1.420)	0.456
>4.8	2.445 (1.943–3.076)	**<0.001**	1.282 (0.993–1.656)	0.056
LODDS				
LODDS1	1		1	
LODDS2	2.420 (1.935–3.028)	**<0.001**	2.266 (1.740–2.950)	**<0.001**
LODDS3	5.222 (4.198–6.496)	**<0.001**	3.869 (2.843–5.266)	**<0.001**

The bold font represents *p* < 0.05, indicating a statistically significant variable.

### 3.3 Establishment of the nomogram

**FIGURE 3:**
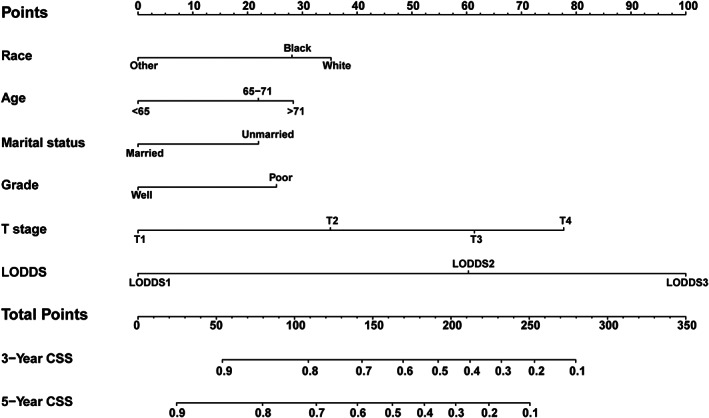
Nomogram predicting CSS of the postoperative patients with GCA.

**FIGURE 4:**
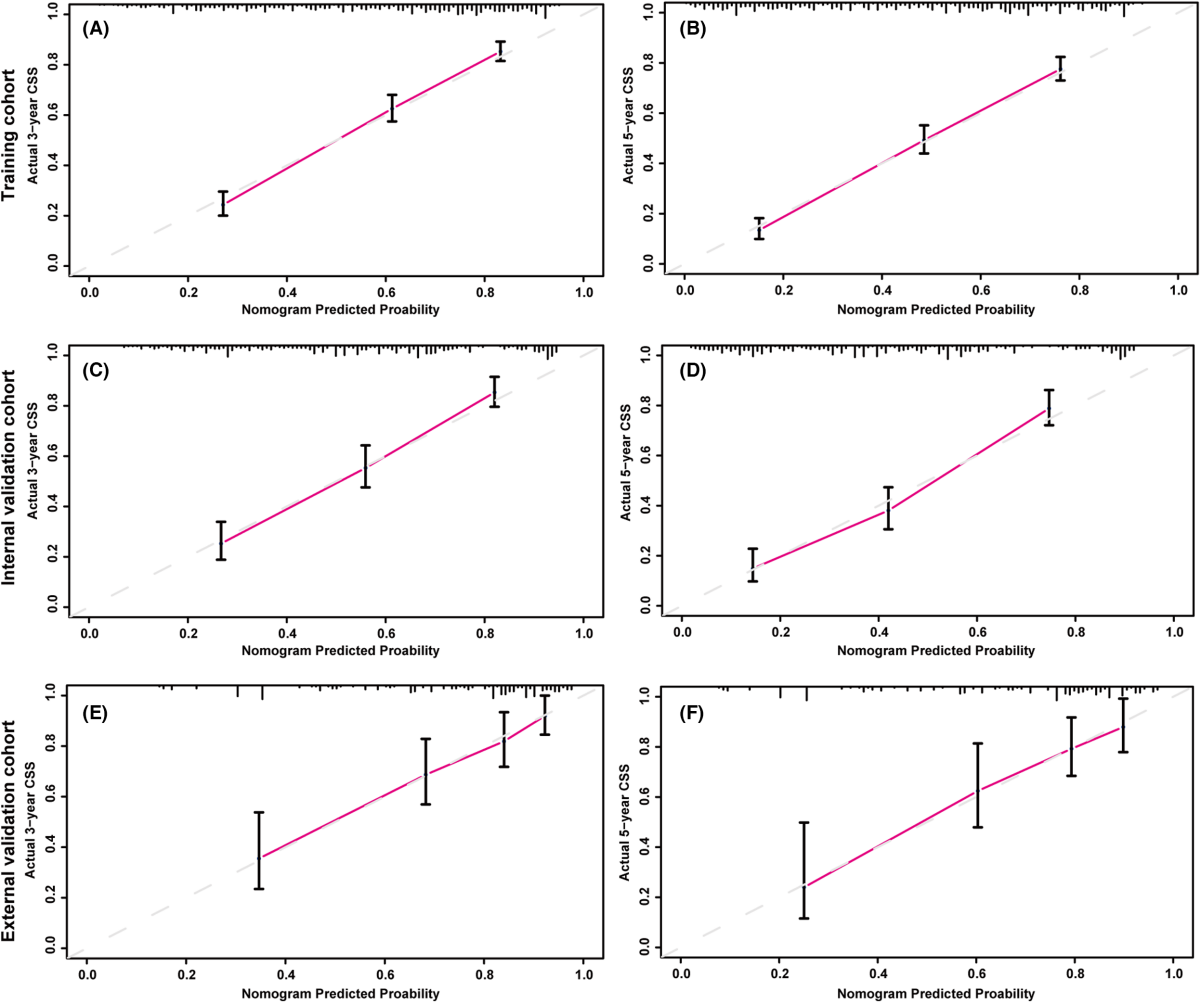
Calibration curves of the nomogram for predicting 3‐year CSS (A) and 5‐year CSS (B) in the training set; the calibration curves of the nomogram for predicting 3‐year CSS (C) and 5‐year CSS (D) in the internal validation set; and the calibration curves of the nomogram for predicting 3‐year CSS (E) and 5‐year CSS (F) in the external validation set.

**FIGURE 5:**
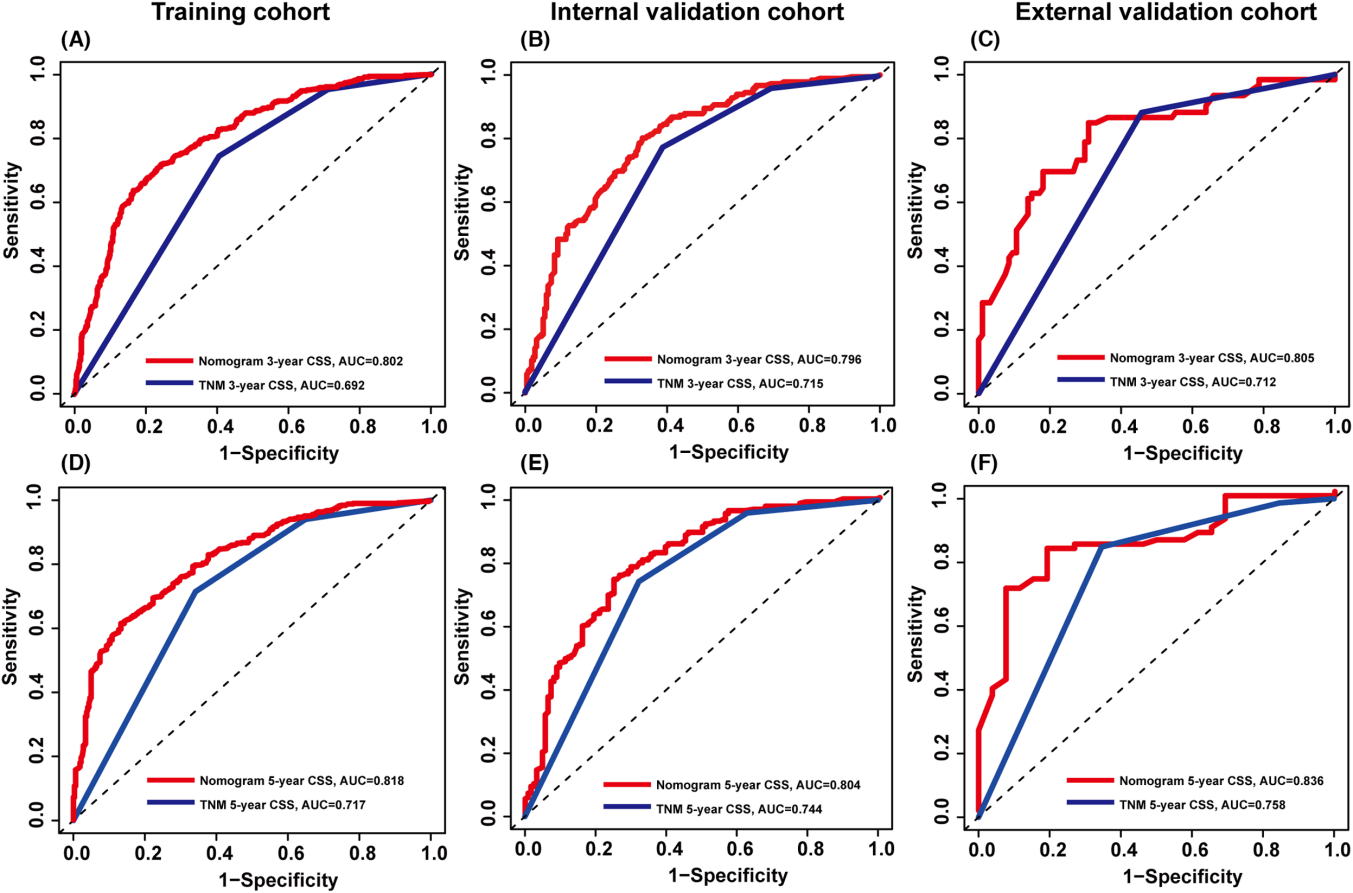
Time‐dependent ROC curves were used to test the predictive power of the 3‐year CSS and 5‐year CSS in the training se (A and D); the internal validation set (B and E); and the external validation set (C and F), respectively.

**FIGURE 6:**
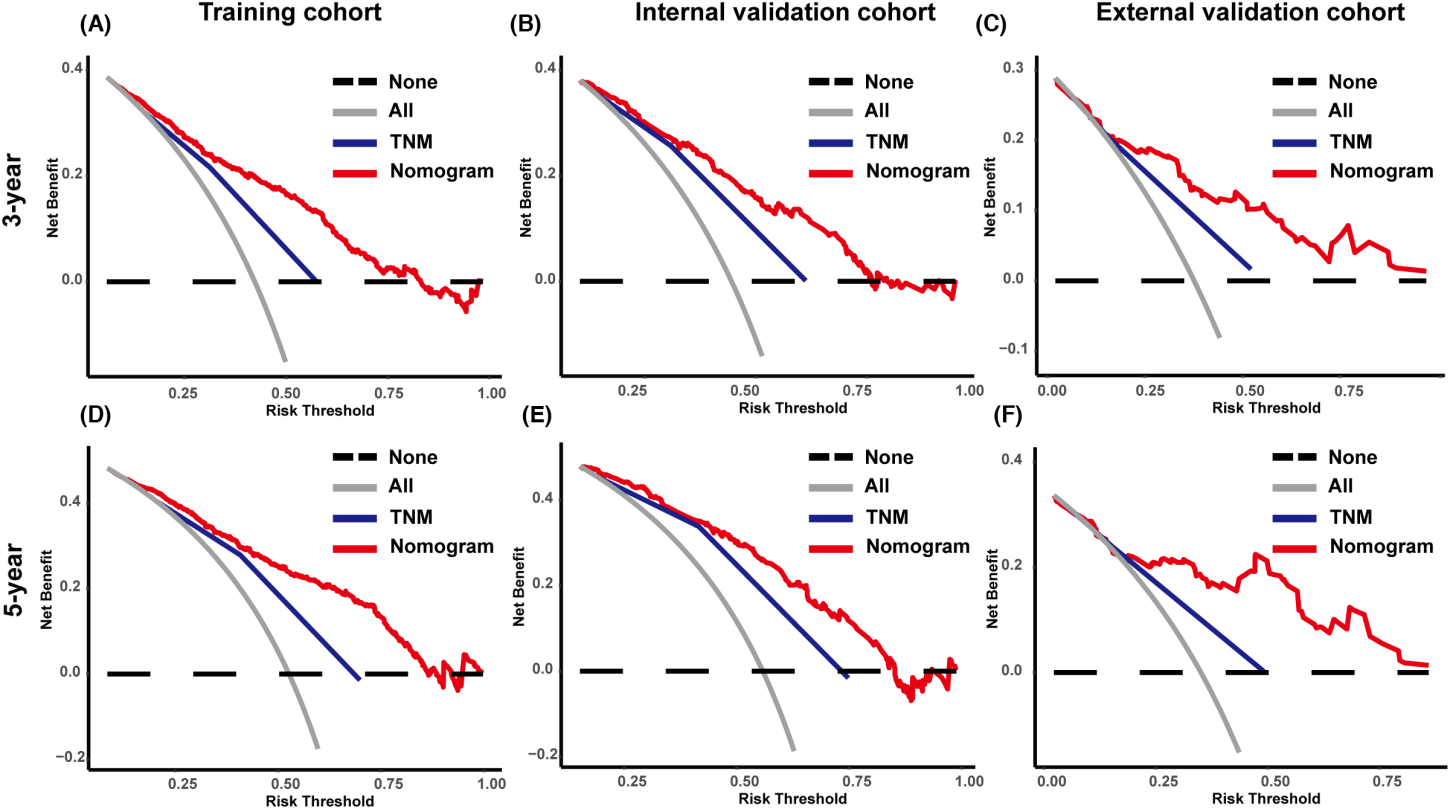
DCA of the nomogram and TNM stage 3‐year CSS and 5‐year CSS of the training (A and D), internal (B and E), and external cohorts (C and F), respectively.

Based on the results of the multivariate Cox regression analysis, a nomogram was established and is presented in Figure 3. The C‐index values for the training, internal validation, and external validation sets were 0.737 (95% confidence interval [CI], 0.717–0.757), 0.733 (95% CI, 0.702–0.764), and 0.746 (95% CI, 0.689–0.803), respectively. The calibration curves in Figure 4 show that the predicted results are consistent with the observed results. As shown in Figure 5, the tdROCs showed that the AUC values of the nomogram in the 3‐ and 5‐year groups were 0.802 (95% CI, 0.774–0.830) and 0.818 (95% CI, 0.789–0.847) in the training set, 0.796 (95% CI, 0.753–0.839) and 0.804 (95% CI, 0.755–0.853) in the internal validation set, and 0.805 (95% CI, 0.732–0.877) and 0.836 (95% CI, 0.746–0.925) in the external validation set, respectively. In comparison, the AUC values of TNM stage in the 3‐ and 5‐year groups were 0.692 (95% CI, 0.663–0.722) and 0.717 (95% CI, 0.683–0.751) in the training set, 0.715 (95% CI, 0.671–0.758) and 0.744 (95% CI, 0.693–0.796) in the internal validation set, and 0.712 (95% CI, 0.646–0.778) and 0.758 (95% CI, 0.655–0.862) in the external validation set, respectively. The nomogram had superior predictive ability compared to that of the TNM stage alone (*p* < 0.05). Furthermore, the DCA curves in Figure 6 show that the nomogram for cancer‐CSS provides significant clinical benefits. In summary, the predictive model based on the aforementioned factors had a strong predictive power for CSS with high accuracy and clinical utility in patients who underwent radical surgery for GCA. This was supported by the results of various statistical analyses such as the –index, calibration curve, ROC, and DCA.

### 3.4 Risk stratification based on the nomogram

**FIGURE 7:**
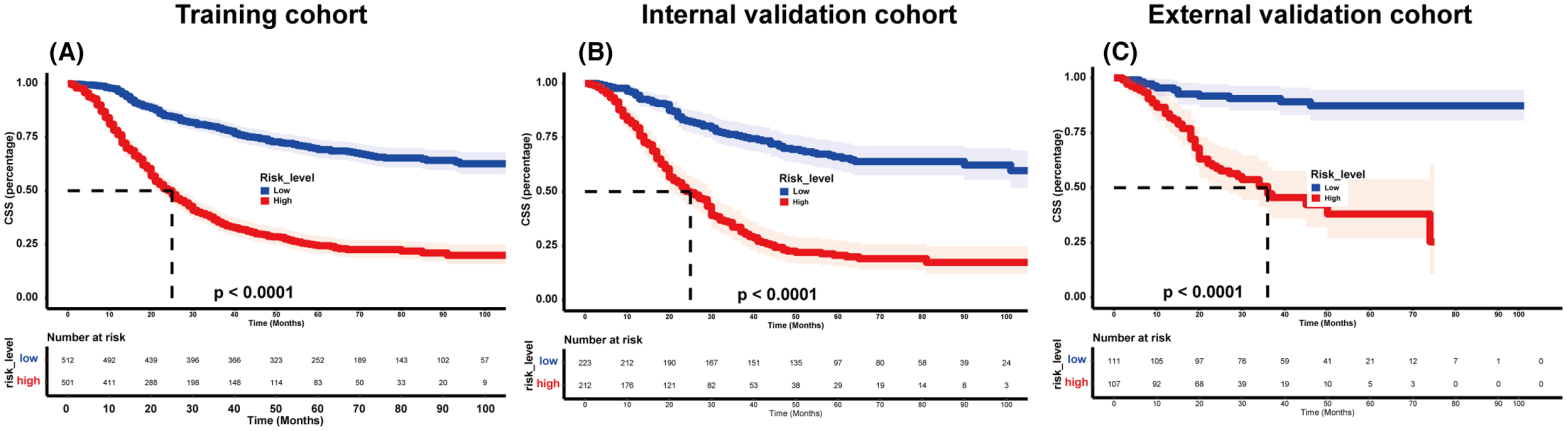
Kaplan–Meier curves for the patients in the low‐ and high‐risk groups based on the risk scores. (A) training cohort; (B) internal validation set; and (C) external validation set.

**FIGURE 8:**
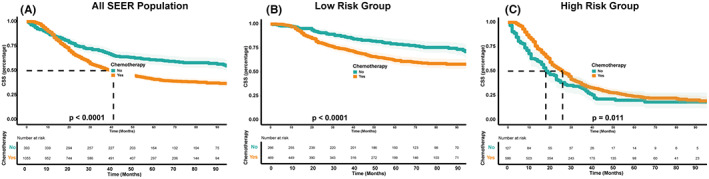
Effect of chemotherapy on the survival in the total population (A), low‐risk group (B), and high‐risk group (C).

The risk score was calculated using the nomogram, and patients were categorized into low‐risk and high‐risk groups based on the median value as the cutoff point. The Kaplan–Meier plot (Figure 7) showed that patients in the low‐risk group had a significantly better prognosis than those in the high‐risk group (*p* < 0.001). Further analysis (Figure 8) revealed that chemotherapy was beneficial only in the high‐risk group, as identified by our model. This suggests that our model can aid physicians in identifying high‐risk patients who may benefit from chemotherapy, allowing for personalized treatment plans.

## 4 DISCUSSION

Currently, mainstream studies consider the EGJ to be a region that is separate from both the esophagus and the stomach.^3^ Given that GCA is a type of malignant tumor occurring in the EGJ that has caused the death of millions of individuals, this disorder warrants further studies. Surgical resection is undoubtedly the most important treatment approach for GCA, with a 5‐year survival rate of 43–49%.^17^ However, despite advancements in treatment, the survival rates for postoperative patients with GCA remain below desired levels, and many experience recurrence and death each year. While the TNM staging system is widely used as a prognostic assessment tool to guide postoperative treatment protocols,^18^ it has limitations in terms of specificity and does not consider important patient factors such as age, sex, and marital status. As a result, the predictive ability of TNM staging may not be sufficiently accurate. In contrast, nomograms are one of the most widely used forecasting tools that can comprehensively consider multiple factors, including clinical pathology and demographic characteristics. This is why our research study focused on developing a nomogram for predicting CSS in postoperative patients with GCA. Previous studies have reported different prognostic models for adenocarcinomas of the EGJ.^5^, ^19^, ^20^ However, most of the previously built models lacked external validation, which reduced their applicability. Therefore, we aimed to construct a predictive model for the prognosis of postoperative patients with GCA at stages I–III GCA and enhance the reliability of the model through validation in an external population.

Our study aimed to construct a nomogram that accurately predicts the prognosis of postoperative patients with GCA at stages I–III based on a multivariate analysis. The C‐index and AUC values were both greater than 0.71, indicating favorable discrimination of the nomogram. Compared to the TNM staging system alone, the nomogram model had a higher accuracy, as shown by the AUC value. The DCA curve was used to analyze the clinical benefits of the model, and the results suggested that the nomogram had a high net clinical benefit. Further statistical analyses showed that the nomogram model had more advantages than the TNM staging system. The use of our nomogram model aligns with the current principles of personalized treatment. Finally, the applicability of the model was verified using an external population.

Although chemotherapy is widely used in the treatment of GCA, our study found that it did not have a positive therapeutic effect on patients in this study.^21^ However, the CLASSIC trial established the benefits of adjuvant capecitabine and oxaliplatin in patients with GC/AEG who underwent surgery.^22^ Based on the nomogram score in this study, we stratified the study population into high‐risk and low‐risk groups. We observed that chemotherapy had a positive therapeutic effect only in the high‐risk group. This indicates that the nomogram‐based identification of high‐risk groups is accurate and can guide clinicians in formulating personalized chemotherapeutic regimens. As for the low‐risk population, the results of our study suggest that chemotherapy may not provide significant therapeutic benefits. Therefore, overtreatment should be avoided to prevent unnecessary adverse effects and improve the patient's quality of life.

In the multifactor analysis, race, age, marital status, histological grade, T stage, and LODDS were determined to be independent prognostic factors. It has been widely reported that older age is a poor prognostic factor in cancer patients.^23^, ^24^ For instance, elderly patients often have functional impairments, malnutrition, and comorbidities that prompt physicians to choose less aggressive treatments or shorten the course of treatment, which in turn affects the treatment outcomes.^25^ In contrast, it has been suggested that younger patients may have a better tolerance to the adverse effects of treatment, including myelosuppression after chemotherapy, compared with that in elderly patients. Additionally, in this study, unmarried patients were more prone to anxiety and had greater stress burdens than married patients, thereby reducing their immune capacity and affecting their metabolic balance, resulting in decreased survival.^26^, ^27^ In addition, unmarried patients had poorer compliance with treatment and received the treatment later, which may also be a reason for their poor prognoses.^28^

Zhu et al. observed that White patients had the highest risk of GCA compared with that in the other ethnic groups, which is consistent with our findings.^7^ In prior studies, race‐related differences in morbidity and outcomes have been attributed to obesity and unequal incomes.^29^, ^30^ In addition, lymph node metastasis has been found to have a significant impact on postoperative outcomes, including long‐term survival and postoperative adjuvant therapy administration.^31^ The results of our study showed that LODDS was a more significant prognostic factor than the conventional N stage in assessing postoperative risk factors for GCA. This is consistent with results of previous studies, suggesting that more attention should be paid to the LODDS of postoperative patients rather than solely focusing on the number of positive lymph nodes.^32^, ^33^, ^34^, ^35^ Additionally, tumors with poor differentiation are generally more aggressive and have a higher likelihood of recurrence and distant metastasis, requiring close monitoring of this patient group.^36^

Compared with the study by Guo et al.,^5^ our study included more variables. Additionally, we conducted an external validation, which enhanced the reliability of our model. To the best of our knowledge, this is the first nomogram to predict the survival of postoperative patients with GCA with both internal and external population validation. However, our study has some limitations. First, important clinical information was lacking from the SEER database. Smoking, alcohol consumption, body mass index, diet, performance, and family history are of great significance in the prognostic evaluation of malignant tumors. In addition, this study did not include molecular or genetic information that is used in routine clinical treatments, such as *EGFR* mutations and Her‐2 expression. Additionally, the SEER database lacks information on specific regimens of chemoradiotherapy, targeted therapy, and immunotherapy, which could have affected the results of our study. Another limitation of our study is that it was retrospective in nature, and thus, a prospective study is necessary to further validate our findings. This will be the focus of our subsequent studies.

## 5 CONCLUSIONS

In conclusion, we utilized clinical data from the SEER database to identify factors associated with survival in postoperative patients with GCA at stages I–III. Subsequently, we developed a nomogram that accurately predicted CSS in patients with GCA who underwent radical surgery. Our findings indicate that the nomogram outperforms TNM staging in terms of predictive power and may provide greater clinical benefits for patients with GCA after radical surgery.

## AUTHOR CONTRIBUTIONS

**Lei Wang:** Conceptualization (lead); methodology (lead); writing – original draft (lead). **Jingjing Ge:** Conceptualization (equal); methodology (equal); writing – original draft (equal). **liwen feng:** Data curation (supporting); visualization (supporting). **Zehua Wang:** Data curation (supporting); visualization (supporting). **Wenjia Wang:** Data curation (supporting); visualization (supporting). **Huiqiong Han:** Conceptualization (equal); writing – review and editing (equal). **Yanru Qin:** Conceptualization (lead); writing – review and editing (lead).

## CONFLICT OF INTEREST STATEMENT

The authors have no conflict of interest to declare.

## ETHICS APPROVAL STATEMENT

The First Affiliated Hospital of Zhengzhou University's Medical Ethics Committee examined and approved this study involving human participants (2023‐KY‐0019‐001).

## PATIENT CONSENT STATEMENT

As this was a retrospective study, no written informed consent was required.

## Data Availability

The datasets utilized in this study can be accessed upon reasonable request to the corresponding author.
